# Supplementary material for: Causal Relationship between Aging and Anorexia Nervosa: A White-Matter-Microstructure-Mediated Mendelian Randomization Analysis
Source: Biomedicines. 2024 Aug 16;12(8):1874. doi: 10.3390/biomedicines12081874 (PMC11351342; doi:10.3390/biomedicines12081874)
Supplement: Supplementary file 1 [file biomedicines-12-01874-s001.zip › Supplementary Figure/Figure S8/Leaveoneout plot of MR analysis for TL to ubm-b-1613.pdf]

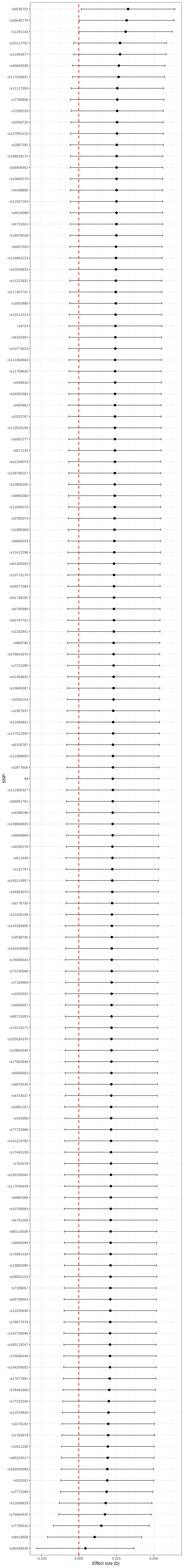
rs6536702
rs35640778
rs1291143
rs10112752
rs11991877
rs45604339
rs117630647
rs11117354
rs7790856
rs7099229
rs2056726
rs137901416
rs2967355
rs188918174
rs59409453
rs10840270
rs4498805
rs11557154
rs4616688
rs4731541
rs16978028
rs6007020
rs116863223
rs12925933
rs11212631
rs117407747
rs3093888
rs12412214
rs4724
rs8102497
rs10774624
rs112394943
rs11769630
rs939916
rs34550383
rs450962
rs1023767
rs113525195
rs6587577
rs871134
rs41269079
rs139795227
rs10905255
rs9955360
rs11085072
rs3785074
rs1985369
rs9600019
rs11412296
rs61405042
rs10773176
rs28577594
rs61748181
rs6790988
rs55747751
rs1332941
rs869785
rs376641875
rs7221585
rs41304832
rs10845387
rs2555104
rs1957937
rs11584821
rs117512405
rs8105767
rs12369950
rs2977608
All
rs111950327
rs56061761
rs9398196
rs139669835
rs6659669
rs4530278
rs611646
rs131797
rs145114957
rs28363070
rs6776756
rs11426156
rs143190905
rs2538745
rs142426306
rs76065543
rs73730598
rs7164950
rs1003322
rs4695407
rs66731853
rs76219171
rs202034370
rs10805346
rs17803849
rs6669563
rs9878436
rs4743037
rs3891167
rs429358
rs77732866
rs141214782
rs17445108
rs762678
rs150150565
rs117034449
rs6881568
rs10768683
rs6751209
rs80116508
rs9940099
rs73581419
rs13062095
rs28502153
rs7209057
rs56799554
rs13230646
rs79977579
rs142730696
rs185174247
rs76666449
rs144204502
rs17677991
rs78491606
rs77231040
rs11579626
rs2276182
rs2763979
rs1611236
rs80324517
rs182059586
rs932002
rs7772289
rs11699829
rs75664430
rs7705526
rs9419958
rs35446936
SNP
-0.025
0.000
0.025
0.050
Effect size (b)
